# Mean total macular volume measurement using optical coherence tomography in already diagnosed primary open angle glaucoma patients

**DOI:** 10.12669/pjms.41.3.10102

**Published:** 2025-03

**Authors:** Hafiz Muhammad Ahmad, Muhammad Younis Tahir, Muhammad Ajmal Chaudhary, Razaullah Khan

**Affiliations:** 1 Hafiz Muhammad Ahmad, FCPS Consultant Ophthalmologist, Quaid-e-Azam Medical College, Bahawalpur, Pakistan; 2 Muhammad Younis Tahir, FCPS, FVR Associate Professor Ophthalmology, Quaid-e-Azam Medical College, Bahawalpur, Pakistan; 3 Muhammad Ajmal Chaudhary, FCPS, FPO Associate Professor Pediatric Ophthalmology, Sheikh Zayed Medical College, Rahim Yar Khan, Pakistan; 4 Razaullah Khan, FCPS, FVR Assistant Professor Ophthalmology, Sheikh Zayed Medical College, Rahim Yar Khan, Pakistan

**Keywords:** Glaucoma, Primary open angle glaucoma, Total macular volume, Optical coherence tomography

## Abstract

**Objective:**

To measure the mean total macular volume (MTMV) in primary open angle glaucoma (POAG) patients using optical coherence tomography (OCT).

**Methods:**

This descriptive cross sectional study was carried out in the department of ophthalmology, Bahawal Victoria Hospital, Bahawalpur from October 2021 to April 2022. A total of 30 patients with POAG, of either gender, between the ages of 20-60 years were included. Patients with secondary glaucoma, having previous intraocular surgery, patients with history of trauma, patients with high myopia and macular disease were excluded. After taking systemic and ophthalmic history, examination of anterior and posterior segments was carried out including best corrected visual acuity (BCVA), intraocular pressure (IOP), central corneal thickness (CCT), gonioscopy, dilated fundus examination of optic nerve and retina, and 30-2° visual field (VF) analysis. OCT was carried out in each patient to measure RNFL thickness and MTMV.

**Results:**

Mean age in our study was 45.50 ± 8.20 years. Majority of these patients 22 (73.33%) were between 41-60 years of age. Among these 30 patients, 20 (66.67%) were male and 10 (33.33%) were females with a male to female ratio of 2:1. MTMV in these POAG patients using OCT was 6.30 ± 1.09 mm^3^.

**Conclusion:**

Our study concluded that POAG patients have a reduced MTMV and it can be easily measuresd by a non-invasive technique using OCT. MTMV loss is a useful indicator of documenting glaucoma and monitoring its progression in POAG patients.

## INTRODUCTION

Glaucoma is a neurodegenerative^1^ disorder of progressive degeneration of retinal ganglion cells (RGCs) and retinal nerve fiber layers (RNFL)^2^ leading to visual disability, blindness and poor quality of life. This RGCs degeneration is also associated with increased intraocular pressure (IOP). Glaucoma with all its types is considered among one of the main causes of irreversible blindness^3^ and is a major public health problem^3^ in the whole world. About 57.50 million people are suffering from primary open angle glaucoma (POAG) worldwide with 02.20% prevalence globally. In Europe, total prevalence is 02.51% and 07.8 million people were affected by POAG.^3^

Previously, Quigley and Broman reported worldwide glaucoma prevalence estimation in 2010 and 2020 with a main focus from European population.^4^ Prevalence of glaucoma is now increasing with the increase in world population particularly from Asian region. Projected increase of glaucoma individuals between the ages of 40 to 80 years is from 76 million in the year 2020 to 111.80 million by the year 2040.^4^ Medical and surgical management is required in all glaucomatous patients to control the IOP. Non-ophthalmic factors like daily coffee intake, obesity, migraine, asthma, smoking^5^ etc and certain systemic problems like arterial hypertension / hypotension, ischemic heart disease and peripheral vasospasm^6^ are responsible for progression of glaucoma, despite good IOP control. A careful evaluation and management of all these risk factors is required to delay glaucomatous damage and prevent blindness in these patients.^5^,^6^

As the glaucoma has variable nature with gradual progression, its stage detection and monitoring are important steps in the management. About half of the RGCs with high cell density are located in macula and this macular area is the best site for the detection of early glaucoma (EG) damage. In this area, 04-06 layers of ganglion cells alongwith RNFL constitutes about 30 to 35% of macular thickness (MT), so loss of these macular ganglion cells in retina results in RNFL thinning. Studies showed that MT and volume are decreased in glaucoma patients due to RGCs degeneration which also correlates with RNFL thinning and visual field (VF) defects.^7^ Thus, measuring the indices of MT and volume is a good indicator of the structural changes that occur in glaucoma. Optical coherence tomography (OCT) is an accurate, convenient method of imaging for diagnosing and monitoring of retinal diseases in routine clinical practice and it has gained much importance in glaucoma assessment.^8^ OCT provides a tomographical three dimensional (3-D) analysis with quantitative and reproducible measurements of optic nerve head (ONH), macular and RNFL parameters by using infrared light.^9^

Gradual and progressive degeneration of RGC complex is the hall mark of POAG. VF defects on standard automated perimetry are seen when structural damage up to 40% has already been occurred. To detect early structural changes and their progression in the macular area, RNFL and ONH is very crucial in the management of POAG. The assessment of MTMV has gained increasing attention in glaucoma diagnosis and management and it can serve as a biomarker for detecting glaucomatous changes in the macula. Most of the previous studies had focused on RNFL thickness or ganglion cell complex (GCC) analysis, leaving a gap in understanding the role of MTMV as a diagnostic tool. Investigating MTMV can have additional diagnostic value, particularly in cases where RNFL or GCC measurements are relatively less reliable. This study aimed to measure the MTMV in POAG patients using OCT. Previously no local data is available on this topic, so our study will provide the local statistics. The results of our study will provide clinicians an evidence supporting the use of macular volume analysis in glaucoma care. Our study will enhance the knowledge of ophthalmologists and will add information to the existing knowledge and food for thought for further research. On the basis of our study results, educational and public health programs can be arranged on national levels to convince our public for regular monitoring of glaucoma to prevent glaucomatous damage and blindess in future.

## METHODS

This descriptive cross sectional study was carried out in the department of ophthalmology, Bahawal Victoria Hospital, Bahawalpur from October 2021 to April 2022. A total of 30 patients were included by non-probability consecutive sampling technique with 95% confidence level, 0.15 margin of error and taking MTMV as 6.18 ± 0.39 mm^3^.^7^

### Ethical Approval:

The ethical review board granted approval for this proposal (No. 1208 / DME / QAMC, Dated: 28-09-2021).

### Inclusion & Exclusion Criteria:

Patients of either gender, with POAG of > 03 months, between the ages of 20-60 years were included. Patients with secondary glaucoma, having previous intraocular surgery, patients with history of trauma, patients with high myopia and macular disease were excluded.

The entire procedure was explained to all patients after obtaining the informed consent. After taking systemic and ophthalmic history, examination of anterior and posterior segments was carried out including visual acuity (VA), best corrected visual acuity (BCVA), slit lamp examination, IOP measurement by applanation tonometery with its correction according to central corneal thickness (CCT), CCT by ultrasound pachymetery, anterior chamber angle examination by three mirror gonioscopy lens, dilated fundus examination of optic nerve and retina using +90 diopter lens, and 30-2° visual field (VF) analysis by automated perimetry. A prototype OCT 3000 was carried out in each patient to measure RNFL thickness and MTMV. Data was recorded on an already designed questionnaire.

Patients having untreated IOP of >21 mmHg on three measurements on different days, having open drainage angles on gonioscopy, having typical optic disc damage with glaucomatous cupping, loss of neuroretinal rim and with confirmed glaucomatous VF defects on two previous reliable VF examinations were labelled as POAG patients.

### Statistical Analysis:

Data was analysed with the Statistical Package for Social Science (SPSS) version 20.0. Mean and standard deviation (SD) were used to represent the quantitative variables like age and MTMV while frequency and percentage were used to represent the qualitative variables like gender. Effect modifiers like age, gender were controlled through stratification and after stratification ‘t’ test was used to measure their effect on MTMV. A significant P-value was considered as ≤ 0.05.

## RESULTS

**Fig.1:**
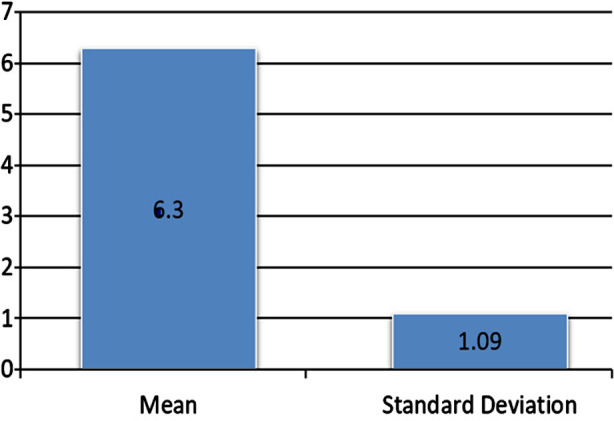
MTMV in POAG eyes using OCT.

Mean age in our study was 45.50 ± 8.20 years with an age range from 20 to 60 years. Majority of the patients 22 (73.33%) were between 41 and 60 years of age as shown in Table-I. Among these 30 patients, 20 (66.67%) were male and 10 (33.33%) were females with a male to female ratio of 2:1. The mean RNFL thickness of these patients was 48.56 ± 9.46 µm. MTMV in these POAG patients using OCT was 6.30 ± 1.09 mm^3^ as shown in Fig.1. Stratification of MTMV according to age groups and gender is shown in Table-II & III respectively.

**Table-I:** Patient distribution according to the age (n=30).

Age (in years)	No. of Patients	%age
20-40	08	26.67
41-60	22	73.33
Total	30	100.0

Mean ± SD = 45.50 ± 8.20 years.

**Table-II:** MTMV stratification according to age groups.

Age groups	Total macular volume		P-value

	Mean	SD	
20-40	6.75	1.17	0.176
41-60	6.14	1.04	

**Table-III:** MTMV stratification according to gender.

Gender	Total macular volume		P-value

	Mean	SD	
Male	6.20	1.01	0.486
Female	6.50	1.27	

## DISCUSSION

We conducted this study to measure the MTMV in POAG patients using OCT. Our results showed that POAG patients have reduced MTMV which is an important biomarker for detecting glaucomatous damage in the macula. POAG, a silent killer of human sight, has no appearent symptoms and signs in its early stage.^10^ In its advanced stage, glaucoma patients are symptomatic having only central vision also called tunnel vision^11^ and later on irreversible loss occurs leading to permanent blindness. IOP measurement, VF examination, cup-disc ratio (CDR) recording and OCT of macular area, RNFL and ONH are important for through diagnostic analysis of glaucoma. OCT is a non-invasive optical biopsy^11^ of the retina to assess optic nerve structure and it is used for detecting the glaucomatous damage occuring over time.^12^

In one study, the MTMV in POAG group and control group was 6.18 ± 0.39 mm^3^ and 6.6 ± 0.17 mm^3^ respectively. This difference of 0.42 among both group means was statistically significant (p < 0.05).^7^ Schulze et al^13^ evaluated the diagnostic ability of retinal GCC, MT, RNFL and ONH parameters with Fourier-domain (FD) OCT in patients with POAG, ocular hypertension (OHT) and in normal subjects. The parameters which have best diagnostic ability in glaucoma patients versus normal subjects were CDR, RNFL thickness and retinal GCC global loss volume. Sung MS et al^14^ evaluated the diagnostic role of MT and RFNL thickness for diagnosing preperimetric and EG in their study. They found almost similar changes in MT which correlated with RNFL thickness among glaucomatous patients and concluded that these parameters can be used to detect subtle structural changes in these patients. Um TW et al^15^ in a study, showed that hemifield MT asymmetry can help in EG diagnosis by showing similar diagnostic value compared to RNFL thickness. Results of our study are comparable to all these studies highlighting that measurement of MTMV using OCT in an important indicator to document and monitor glaucoma progression in POAG patients.

The findings of our study are in strong agreement with the findings of the Sullivan-Mee et al^16^ study, which found that RNFL thickness and intra eye MT asymmetry had superior diagnostic performance and these two parameters were important in EG diagnosis. Silverstein E et al conducted a study^17^ on 80 eyes of 80 children, 37 eyes were glaucomatous and 43 eyes were non-glaucomatous, with a conclusion that OCT is a useful tool in children as well particularly for children who are not cooperative for VF studies. They found that in glaucomatous eyes macular nerve fiber layers, ganglion cell layers, inner plexiform layers and peripapillary RNFLs are thinner in comparison to non-glaucomatous eyes as proposed by our study.

Africa and Asia have highest glaucoma prevalence and Asia represents 47% of all glaucomas^18^, out of which 53.4%^19^ are POAG cases. In Pakistan, glaucoma is a third leading cause of irreversible blindness and accounts for 07.1% of cases.^20^ In Pakistani population, more than 1.8 million people are suffering from glaucoma and half of them have permanent visual loss before diagnosis.^11^ Arshad H et al. from Pakistan conducted a study and showed that most of glaucoma patients (82.2%) present at an AG stage.^21^ In developing countries like Pakistan, the primary method of glaucoma detection is optic nerve examination. OCT assessment is always helpful when available.^22^ Our study revealed that MTMV is an important indicator of documenting glaucoma and monitoring its progression. So, OCT should always be used to diagnose and monitor glaucoma patients. Our this study will add to the already published national research evidence on this topic.

### Limitations:

A limited sample size with limited follow-up duration is a possible limitation of our study. Further prospective, long-term researches with large sample size on diagnostic power of OCT in glaucoma are required to complement our findings and to formulate the best way to use these parameters into clinical practice in glaucoma patients. Ophthalmologists should be aware of OCT scan artifacts related to the scanners, ophthalmic technicians and related to disease itself that every reduced MTMV and RNFL thickness is not glaucoma.

## CONCLUSION

Our study concluded that POAG patients have reduced MTMV. MTMV can be easily measuresd by a non-invasive technique using OCT. MTMV can be used in addition to RNFL thickness in detecting and monitoring glaucoma, particularly in patients with disk abnormalities like peripapillary atrophy in which RNFL thickness is not reliable and MTMV can be relied upon. MTMV loss is a useful indicator of documenting glaucoma and monitoring its progression.

### Recommendations:

We recommend that educational and public health programs should be arranged at provincial and national levels to convince our public for regular monitoring of glaucoma by OCT to prevent glaucomatous damage and blindess in future. In Pakistan, OCT is now mostly available in almost all hospitals and should be used to diagnose and monitor glaucoma patients.

### Authors’ Contribution:

**HMA:** Did data collection, Statistical analysis and manuscript writing.

**MYT:** Did data collection, interpretation of data and manuscript writing.

**MAC:** Conceived, designed and did editing of manuscript.

**RK:** Did review, critical analysis, and final approval of manuscript.

All authors have read, approved the final version and are accountable for the integrity of the study.
